# Dispositional employability and self-regulation in antisocial and prosocial personalities: different contributions to employability

**DOI:** 10.1186/s40359-023-01037-1

**Published:** 2023-01-09

**Authors:** Elena Lisá, Michaela Valachová

**Affiliations:** Faculty of Social and Economic Sciences, Comenius University in Bratislava, Mlynské Luhy 4, 821 05 Bratislava, Slovakia

**Keywords:** Employability, Dark triad, Light triad, Honesty-humility, BAS drive

## Abstract

The manuscript is based on the dispositional approach of employability, dispositional personality trait theories (dark triad, light triad, HEXACO honesty-humility), and reinforcement sensitivity theory. The facet-focused analysis allowed a more targeted interpretation of the results about the contribution of dark/light personalities and self-regulation for employability and a deeper understanding of practical implications. We analyzed the mediating effect of the behavioral activating system (BAS drive) on antisocial and prosocial traits in predicting employability. The convenient research sample consisted of 343 students. Participants completed: The short dark triad, light triad, honesty-humility, dispositional employability, and BIS/BAS. Dark traits explained 17.5% of work/career resilience, 12% of work identity, 6.4% of career motivation, and 6.6% of openness to changes at work. Narcissism explained 20% of work/career resilience. Prosocial traits explained 19.7% of work/career resilience, 16.8% of work identity, 11.8% of career motivation, and 5.3% of openness to changes at work. Modesty explained 10% of career motivation variance. BAS drive mediates predictions of employability by prosocial and antisocial traits. Demanding attention from others and focusing on making a good impression are effective tools for employability. Prosocial traits significant for BAS drive-activated participants (believing in the goodness of people and avoiding fraud and corruption) can be supported in organizations by providing career growth opportunities.

## Introduction

In the current pandemic situation, there is enormous pressure on employees to adapt more effectively to the changing environment. Dispositional employability refers to the innate adaptability to change in work or career settings. Fugate and Kinicki [1] stress the importance of reactive and proactive traits of dispositional employability. The current study aims to explore factors that may play a critical role in enhancing employability so appropriate interventions can be targeted at young employees. Prosocial and antisocial personality traits are examined as predictors of employability in relation to self-regulation factors. It is generally assumed that prosocial traits are essential for career development; however, the research on this is limited. Prosocial competencies are employability skills sought by employers [2, 3]. Self-regulatory skills also fall into this category [4, 5]. Although employers do not focus on antisocial traits, the latter are often linked to successful careers, specifically to higher salaries and leadership positions [6–9]. Are prosocial, dark traits, and self-regulation equally good at predicting employability? That is the question we attempt to answer in this research study.

Employers wish to have employees with prosocial traits. However, the research does not show connection between prosocial personality traits and career success. On the other hand, we have some evidence about the dark traits and career success. As the dark ones are often successful in their careers, there use certainly some career skills, that are worth to learn.

Our study draws on dispositional personality trait theories (Dark Triad, Light Triad, HEXACO Honesty-Humility) [10–12] and reinforcement sensitivity theory (RST) [13].

Employability includes understanding market requirements and matching them with one's own internal and external resources. It expresses three basic perspectives [14], which cannot be separated from each other: educational and governmental, organizational and individual. The rationale of our research results from the organizational perspective (requirements of employers), with impacts on the individual perspective (prosocial and self-regulatory characteristics of the individual, dispositional employability) in the educational/governmental context (examined on a sample of university students).

As McQuaid and Lindsay [15] reported, the demand factor is included in external employability factors. We will analyze the individual level of employability and related personality characteristics, depending on demands from the external environment of employers. The external environment can not only define the requirements for employability characteristics [2], but can also offer opportunities to develop the dispositional capacity of an individual [14].

## Employability

Employability is a construct that has been researched for almost a century and still needs a uniformly defined theory, definition, or measurement tool. It generally expresses “individual's potential in the labour market,” (p. 145) which is expressed in three strands [16]: (1) personal strengths that promote the individual's employment potential, (2) self-perceived employment opportunities, and (3) job transitions as a realization of potential. The strands are separated units but with components interrelated within and across the strands. Job transitions depend on perceived employment opportunities and personal strengths; employment opportunities depend on personal strengths. That means that employability builds on personal strengths, representing the individual's perspective of employability that can be expressed as competencies [17], employability orientation [18], personality dispositions [1], personal values and protean career attitude [19, 20], or adaptability [21, 22]. This individual level of employability represents the interest of psychology. It is expressed as individual factors [15], micro-level of the individual [23], individual perspective [14], individual strength [24], competence-based view of employability [25].

In the current study, we work with dispositional theories and, therefore, with the dispositional theory of employability. “…the disposition of employability is conceptualized as encompassing both reactive and proactive personal characteristics. This means that in addition to the ability to adapt reactively to known demands, employable individuals tend to have a perpetual readiness for change” [1] (p. 505). Dispositional employability is a multidimensional construct consisting of five dimensions: openness to changes at work; work and career resilience; work and career proactivity; career motivation, and work identity. The construct accounts for personal factors, structural factors, and their interaction [23] because it combines the individual attitude with the work environment.

### Anti/prosocial personality traits and employability

Personality traits have been shown to predict career characteristics in cross-sectional [26] and longitudinal research settings [27]. The dark triad (DT) maps socially aversive traits, lower compassion, high impulsivity, and non-agreeableness [10]. It can be contrasted with prosocial characteristics, such as the light triad (LT) [11] or honesty-humility (HH) [12]. Antisocial personality traits, represented by dark traits, have been broadly examined in relation to personality differences [28], motivation [29], organizational psychology [30], mental and physical health [31, 32]. Dark personalities have previously been found to be counterproductive in organizational settings [33]. People with this type of personality are sources seekers with a great desire for power, prestige, and money [34]. At the other end of the scale are people with high honesty-humility [35]. Source seekers’ characteristics may positively or negatively relate to perceived employability. Narcissism positively predicts perceived employability, whereas psychopathy is a negative predictor, and Machiavellianism is not a significant predictor [36].

Besides concepts such as the dark triad (Narcissism, Psychopathy, Machiavellianism) or dark core [37], there are traits connected to prosocial behavior such as humanism, altruism, empathy (Humanism, Faith in Humanity, Kantianism) [11], and prosocial tendencies (Honesty-Humility) [12]. Honesty-humility relates significantly and negatively to all three dark triad dimensions [38–40] and positively to the humanistically defined traits known as the light triad [11]. Machiavellianism and psychopathy have negative relationships with all three light triad traits, and narcissism correlates significantly with Kantianism only [11].

Prosocial behavior is crucial for cooperation and hence essential in any healthy organization. Surprisingly, agreeableness has been found to negatively predict perceived employability [27]. People low in agreeableness reported higher marketability than people high in agreeableness. On the other hand, prosocial young adults thought they had better future employment prospects [41]. Despite job requirements, the associations between prosocial traits and employability have not been examined sufficiently [27, 36], and the empirical findings are ambiguous.

Based on these findings, we assume hypotheses H1 and H2:

#### H1

Antisocial personality traits significantly predict employability.

#### H2

Prosocial personality traits significantly predict employability.

### Self-regulation in anti/prosocial personality

In reinforcement sensitivity theory [13, 42–45], two systems of neural regulation explain individual motivational orientation. The behavioral inhibiting system (BIS) and behavioral activating (BAS) system determine the extent to which individuals perform optimally when exposed to an aversive stimulus (e.g., punishment) or appetitive stimulus (e.g., reward). For example, if people overreact to punishment (higher BIS), their study/work behaviors will lead to exhaustion and the intention to quit [46]. Conversely, those who overreact to reward (higher BAS) show better career planning predispositions such as career adaptability or optimism and better work/study performance [26, 46]. Reward as an appetitive stimulus is typically found in work settings.

BIS and BAS are biological precursors of temperamental personality traits such as neuroticism and extraversion [47], which empirically relate to light and dark traits [11], giving them specific shared qualities such as drive or impulsivity. BAS is a multi-dimensional factor [48] that includes BAS drive (the motivation to follow one's goals), which is positively associated with all dark traits [29]. People high in BAS drive tend to have issues with self-regulation, impulse control, and engage in more intensive reward seeking, while ignoring the long-term consequences [43, 48]. The research on relationships between prosocial traits and self-regulation is inconsistent. BIS is a biological precursor of neuroticism that has been shown to positively correlate with moral emotions and moral judgments [49], which suggests that prosocial people are afraid of punishment experienced as shame. The light triad has a significant positive relationship with neuroticism [50]. However, honesty-humility relates negatively to BAS, and is not linked to BIS [51]. Recent studies show a positive relationship between BAS drive and prosocial behavior [52]. It appears that BAS drive can benefit both anti- and prosocial [29, 52] personalities in the workplace. BAS drive has been recorded as stimulating dorsomedial striatum activity, which is typically associated with instrumental performance [53]. We can assume that a common motivational source (BAS) explains differences in individual motivational orientation.

### Employability and self-regulation

Personal qualities such as adaptability signal standout employability [54]. Self-awareness and adaptability are considered predictors of employability [4, 55–57]. Job requirements include prosocial behavior or self-regulating skills. In the eyes of employers, learning from feedback, flexibility, and adaptability are the key skills of graduate employability [2]. These qualities require an adequate level of self-regulation.

Adaptability is an essential part of dispositional employability. Dispositional employability is defined as "one's ability to realize job opportunities within and between employers over time" (p. 1) [58]. This is a reference to personal adaptability, which is essential for both employees and employers in today's dynamic work environment. Individuals now have to negotiate a never-ending series of workplace changes and transitions. Employable people are proactively oriented to goal achievement, which means they react to changing environments and create conditions to fit their needs [1]. Dispositional employability facilitates the identification of new opportunities, future changes, and learning possibilities. Dynamic adaptation and specific individual attributes give employable people the energy to realize the opportunities required for success [1]. Active adaptation and energy emanate from an inner source and relate to the neural regulation mechanism of BAS. The BAS is a supporting mechanism for pursuing and achieving goals, for example, employability goals. BAS is the strongest predictor of career adaptability and exploration [59]. Besides five-factor personality traits, BAS uniquely contributes to two significant career/employability variables. Career exploration is defined as “purposive behavior and cognitions that afford access to information about occupations, jobs, or organizations” (p. 192) [60]. Career adaptability is “the readiness to cope with the predictable tasks of preparing for and participating in the work role and with the unpredictable adjustments prompted by changes in work and working conditions” (p. 254) [61]. Therefore, we can assume that BAS positively predicts dispositional employability. Self-regulation as a mediator in predicting staff productivity has proven to be a significant factor in Iranian higher education employees [5].

Based on the above, we formulate H3 and H4.

#### H3

We assume that dark traits significantly predict employability and that BAS drive positively mediates this prediction.

#### H4

We assume that prosocial traits (light triad, honesty-humility) significantly predict employability and that BAS drive positively mediates this prediction.

## Materials & methods

### Participants

The research sample consisted of 343 university students in Slovakia (30.32% men; M_age_ = 22.30 years). Convenient sampling was used, and students participated voluntarily. They responded to the open call for university students on social networks. One-third of them was collected in the psychology department. The rest of them did not refer to the field of study. They all studied at the same university. The online questionnaire consisted of informed consent, followed by the research scales in the exact order given below. We added three control questions to the research items. From the original 353 participants we excluded ten students who gave incorrect answers to these. The other eligibility criteria for inclusion in the research sample were: studying at a Slovak university and speaking Slovak as their first language. We monitored the following demographic variables: age and gender.

### Measures

The BIS/BAS scale [62] consists of 20 items comprising one behavioral inhibition scale (BIS) and three behavioral activation system scales (BAS). The BIS has seven items that assess sensitivity to punishment (e.g., “I worry about making mistakes”; *α* = 0.77; *ω* = 0.78). The BAS has three facets: Reward Responsiveness (five items, e.g., “When I'm doing well at something, I love to keep at it”; *α* = 0.69; *ω* = 0.70), Drive (four items, e.g., “I go out of my way to get things I want”; *α* = 0.83; *ω* = 0.83), Fun Seeking (four items, e.g., “I crave excitement and new sensations”; *α* = 0.78; *ω* = 0.78). The participants responded on a four-point Likert scale ranging from 1 (strongly disagree) to 4 (strongly agree), with average gross score ranging from 1 to 4. The model has an excellent fit to the data *X*^*2*^ (N = 343; *df* = 164) = 323.36; *TLI* = 0.97; *CFI* = 0.98; *RMSEA* = 0.05; *p* < 0.001.

The Short Dark Triad scale [63] measures the antisocial, dark personality traits through 27 items. Nine items measure each of the following facets: Machiavellianism (e.g., “I tend to manipulate others to get my way”; *α* = 0.75; *ω* = 0.76); Narcissism (e.g., “I am an average person”; *α* = 0.73; *ω* = 0.74); and Psychopathy (e.g., “I tend to avoid danger situations”; *α* = 0.73; *ω* = 0.74). Participants responded on a five-point Likert scale ranging from 1 (completely disagree) to 5 (completely agree). CFA of the model showed low values of fit indices [*TLI* = 0.879; *CFI* = 0.889; *RMSEA* = 0.087]. We allowed inter-correlations of items among factors. Results showed adequate data fit indices [*X*^*2*^ (N = 343; *df* = 306) = 776.72; *TLI* = 0.928; *CFI* = 0.937; *RMSEA* = 0.067; *p* < 0.001]. All three traits share emotional deficits [64] and antagonism [65], which could explain inter-correlations of factors and their items.

The Light Triad scale [11] consists of 12 items and is divided into three facets: Faith in Humanity (four items, e.g., “I tend to see the best in people”; *α* = 0.71; *ω* = 0.72), Humanism (four items, e.g., “I tend to applaud the successes of other people”; *α* = 0.69; *ω* = 0.70), and Kantianism (four items, e.g., “I prefer honesty over charm”; *α* = 0.64; *ω* = 0.65). Each facet includes four items rated on a five-point scale, ranging from 1 (very strongly disagree) to 5 (very strongly agree). The three-factor model demonstrated an adequate fit with the data *X*^*2*^ (N = 343; *df* = 249) = 805.32; *TLI* = 0.90; *CFI* = 0.91; *RMSEA* = 0.08; *p* < 0.001.

The honesty-humility scale measures prosocial tendencies derived from HEXACO-PI-R [12]. The scale has 16 items and the Slovak language version was recently verified [66]. It includes four facets: Sincerity (four items, e.g., “I wouldn't use flattery to get a raise or promotion at work, even if I thought it would succeed”; *α* = 0.63; *ω* = 0.63), Fairness (four items, e.g., “I would be tempted to buy stolen property if I were financially tight”; *α* = 0.70; *ω* = 0.74), Greed Avoidance (four items, e.g., “Having a lot of money is not especially important to me”; *α* = 0.81; *ω* = 0.82), and Modesty (four items, e.g., “I am an ordinary person who is no better than others”; *α* = 0.55; *ω* = 0.57). Participants answer on a five-point Likert scale ranging from 1 (strongly disagree) to 5 (strongly agree). The four-factor model showed an excellent fit with the data *X*^*2*^ (N = 343; *df* = 98) = 453.41; *TLI* = 0.98; *CFI* = 0.98; *RMSEA* = 0.05; *p* < 0.001.

The dispositional employability scale [1] has 25 items and measures five dimensions: Openness to Changes at Work (five items e.g., “I can handle job and organizational changes”; *α* = 0.80; *ω* = 0.79), Work/Career Resilience (eight items, e.g., “I feel I am a valuable employee at work”; *α* = 0.84; *ω* = 0.84), Work/Career Proactivity (three items, e.g., “I stay abreast of developments in my industry”; *α* = 0.88; *ω* = 0.88), Career Motivation (three items, e.g., “I have a specific plan for achieving my career goals”; *α* = 0.68; *ω* = 0.68), and Work Identity (five items, e.g., “I define myself by the work that I do”; *α* = 0.78; *ω* = 0.79). Because the research sample consisted of university students, we added “university” to the items Hence the original item, e.g., “I feel I am a valuable employee at work” was expanded to: “I feel I am a valuable employee/student at work/university”. The five-factor model has an adequate fit with the data *X*^*2*^ (N = 343; *df* = 265) = 791.51; *TLI* = 0.98; *CFI* = 0.98; *RMSEA* = 0.08; *p* < 0.001.

### Procedures

Two independent translators translated the BIS/BAS, Light Triad, and Dispositional Employability scales into Slovak. The first step was to perform a confirmation factorial analysis to verify whether the measurement models fit the data. Model fit was assessed using the Comparative Fit Index (CFI) and Tucker-Lewis Index (TLI) and gave acceptable values ˃ 0.90 [67]. Values of ˃ 0.90 represent adequate, and values of ˃ 0.95 good data fit [68]. The Root Mean Square Error of Approximation (RMSEA) was used to assess the model’s absolute fit [69]. RMSEA values of ˂0.08/˂0.05 show adequate/good fit [68]. Subsequently, multivariate regression analyses (mediation analysis) were performed. We used delta method standard errors, bias-corrected percentile bootstrap confidence intervals with 5000 replications, DWLS estimator.

The Kurtosis and Skewness values ranged between − 1 and 1, except for the work/career proactivity facet of employability. Given the abnormal distribution, we excluded the Work/Career Proactivity variable from the analysis. The gender differences in Machiavellianism (t = 2.413; /////*p* = 0.016), psychopathy (t = 4.364; p ˂ 0.001), humanism (t = − 3.003; *p* = 0.003), fairness (t = − 2.901; *p* = 0.004), greed avoidance (t = − 2.164; *p* = 0.031), modesty (t = − 2.011; *p* = 0.045) led us to control for gender. Humanism had the biggest correlation with age (r = − 0.158), indicating that age was not a significant demographic variable. We checked the multicollinearity of the regression models via VIF and Tolerance coefficients. They did not show any multicollinearity issues. The data were analyzed using JASP [70].

The independent explanatory variables were dark traits – DT (indexed as mach – Machiavellianism, narc – narcissism, psych – psychopathy), light traits – LT (indexed as LTfh – faith in humanity, LThu – humanism), and Honesty-Humility – HH (indexed as HHf – fairness, HHg – greed avoidance, HHm – modesty). Dispositional employability – DME (indexed as Eop – openness to changes at work, Emot – career motivation, Eres – work/career resilience, Eid – work identity) was the dependent (explained) variable. BAS drive (indexed as BASd) was the mediator.

All data are available at Figshare.com with reserved https://doi.org/10.6084/m9.figshare.19286159. Research materials are available upon the references used in the methods section. This study’s design and its analysis were not preregistered.

## Results

The descriptive statistics and correlations are presented in Table 1. Of the dark traits, only narcissism positively correlates with all the employability dimensions (correlation coefficients are between 0.250 and 0.376). Conversely, psychopathy does not correlate with the employability dimensions, and Machiavellianism correlates positively only with work identity (r = 0.183). None of the honesty-humility facets correlate with employability openness to changes at work. HH modesty correlates negatively with the rest of the employability facets (correlation coefficients between − 0.182 and − 0.303). HH greed avoidance correlates negatively with work/career resilience (r = − 0.107) and work identity (r = − 0.214). HH fairness correlates positively with career motivation (r = 0.120) and work/career resilience (r = 0.270). HH sincerity correlates negatively with work identity only (r = − 0.171). Turning to the light triad, Kantianism does not correlate with any of the employability dimensions. LT humanism correlates positively with all the employability dimensions (from 0.154 till 0.253). LT faith in humanity does not correlate with career motivation, but the remaining employability dimensions correlate positively from 0.120 to 0.297. BAS drive correlates positively with all the employability dimensions (from 0.292 to 0.523). All HH facets correlate negatively with all dark traits (correlation coefficients from − 0.152 till − 0.558). Light traits correlate negatively with Machiavellianism and psychopathy (correlation coefficients from − 0.156 till − 0.508). Kantianism correlates negatively with narcissism (r = − 0.242).

**Table 1:** Correlations, means, and standard deviations

Variable	1	2	3	4	5	6	7	8	9	10	11	12	13	14	15
1. BASd	–														
2. Mach	0.259***	–													
3. Narc	0.341***	0.253***	–												
4. Psych	0.151**	0.449***	0.387***	–											
5. LTfh	0.191***	− 0.275***	0.066	− 0.192***	–										
6. LThu	0.182***	− 0.168**	0.069	− 0.156**	0.442***	–									
7. LTka	− 0.036	− 0.508***	− 0.235***	− 0.336***	0.302***	0.276***	–								
8. HHs	− 0.021	− 0.409***	− 0.244***	− 0.319***	0.063	0.100	0.481***	–							
9. HHf	0.111*	− 0.333***	− 0.160**	− 0.442***	0.184***	0.209***	0.313***	0.300***	–						
10. HHg	− 0.021	− 0.342***	− 0.356***	− 0.292***	0.076	0.064	0.430***	0.422***	0.314***	–					
11. HHm	− 0.243***	− 0.387***	− 0.562***	− 0.430***	0.128*	0.122*	0.360***	0.401***	0.272***	0.495***	–				
12. Eop	0.292***	− 9.789e− 5	0.250***	0.036	0.235***	0.189***	0.087	0.094	0.029	0.041	− 0.034	–			
13. Emot	0.470***	0.094	0.251***	0.046	0.075	0.154**	− 0.004	− 0.080	0.120*	− 0.061	− 0.246***	0.204***	–		
14. Eres	0.523***	− 0.037	0.376***	− 0.036	0.297***	0.253***	0.054	− 0.029	0.207***	− 0.107*	− 0.182***	0.478***	0.524***	–	
15. Eid	0.425***	0.183***	0.278***	− 0.00	0.120*	0.184***	− 0.043	− 0.171**	0.074	− 0.214***	− 0.303***	0.277***	0.501***	0.518***	–
AM	3.079	3.064	2.690	2.059	3.433	3.911	3.813	3.472	3.441	3.371	3.671	3.859	3.247	3.468	3.600
SD	0.615	0.704	0.727	0.678	0.891	0.762	0.772	0.907	1.038	1.055	0.806	0.702	1.086	0.802	0.777

*p < 0.05; **p < 0.01; ***p < 0.001; Conditioned on variables: age

Table 2 shows significant predictive relationships. Altogether dark traits explain 17.5% of work/career resilience (F = 23.952; p ˂ 0.001), 12% of work identity (F = 15.414; p ˂ 0.001), 6.4% of career motivation (F = 7.780; p ˂ 0.001), and 6.6% of openness to changes at work (F = 7.968; p ˂ 0.001). Narcissism is the strongest predictor; it explains 20% of the variance in work/career resilience. Narcissism and Machiavellianism positively predict employability and negatively predict psychopathy.

**Table 2:** Linear regression (independent variable: dark traits, prosocial traits; dependent variables: employability dimensions)

Predictor	Dependent variable	Unstandardized	SE	Standardized	t	*p*
DT narcissism	Openness to changes at work	0.264	0.055	0.273	4.77	< 0.001
DT narcissism	Career motivation	0.379	0.086	0.254	4.42	< 0.001
DT narcissism	Work/career resilience	0.502	0.059	0.455	8.45	< 0.001
DT narcissism	Work identity	0.327	0.060	0.306	5.49	< 0.001
DT psychopathy	Work/career resilience	− 0.240	0.064	− 0.202	− 3.77	< 0.001
DT psychopathy	Work identity	− 0.261	0.070	− 0.228	− 3.76	< 0.001
DT machiavellianism	Work identity	0.219	0.064	0.198	3.43	< 0.001
HH fairness	Career motivation	0.172	0.057	0.165	3.03	0.003
HH fairness	Work/career resilience	0.160	0.040	0.207	3.97	< 0.001
HH fairness	Work identity	0.121	0.041	0.162	2.99	0.003
HH greed avoidance	Work identity	− 0.088	0.043	− 0.119	− 2.02	0.044
HH modesty	Career motivation	− 0.428	0.072	− 0.318	− 5.95	< 0.001
HH modesty	Work/career resilience	− 0.292	0.051	− 0.293	− 5.74	< 0.001
HH modesty	Work identity	− 0.297	0.056	− 0.308	− 5.29	< 0.001
LT faith in humanity	Openness to changes at work	0.181	0.042	0.229	4.35	< 0.001
LT faith in humanity	Work/career resilience	0.212	0.049	0.235	4.29	< 0.001
LT humanism	Career motivation	0.210	0.075	0.147	2.79	0.005
LT humanism	Work/career resilience	0.139	0.058	0.132	2.39	0.017
LT humanism	Work identity	0.210	0.052	0.206	4.03	< 0.001

Altogether prosocial traits explain 19.7% of work/career resilience (F = 20.709; p ˂ 0.001), 16.8% of work identity (F = 17.031; p ˂ 0.001), 11.8% of career motivation (F = 15.143; p ˂ 0.001), and 5.3% of openness to changes at work (F = 18.932; p ˂ 0.001). HH modesty is the strongest predictor of the prosocial trait and explains 10% of career motivation variance. HH greed avoidance and HH modesty negatively predict employability, while HH fairness, LT faith in humanity, and LT humanism positively predict employability.

HH sincerity and LT Kantianism do not predict any of the employability dimensions. Therefore, they were not included in the subsequent mediation analysis.

**Fig. 1:**
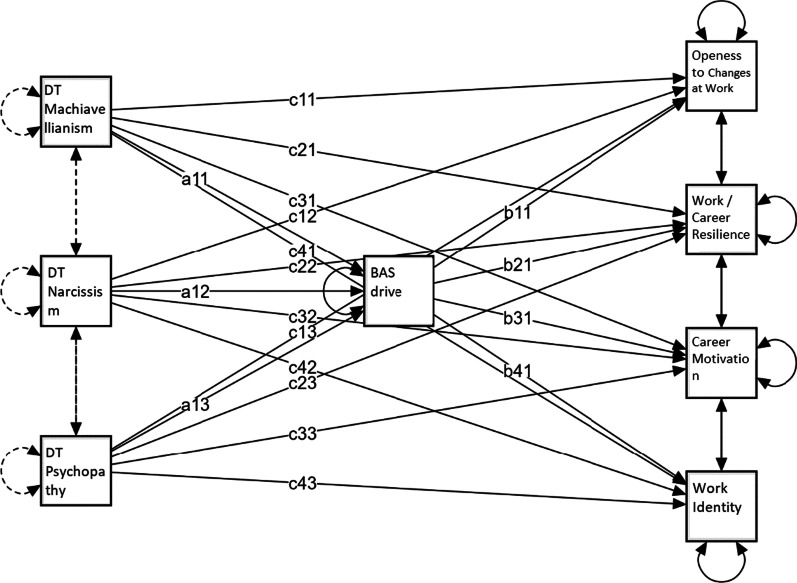
Dark traits predict employability dimensions, with BAS drive mediation (a = direct effect; b = indirect effect; c = total effect)

**Fig. 2:**
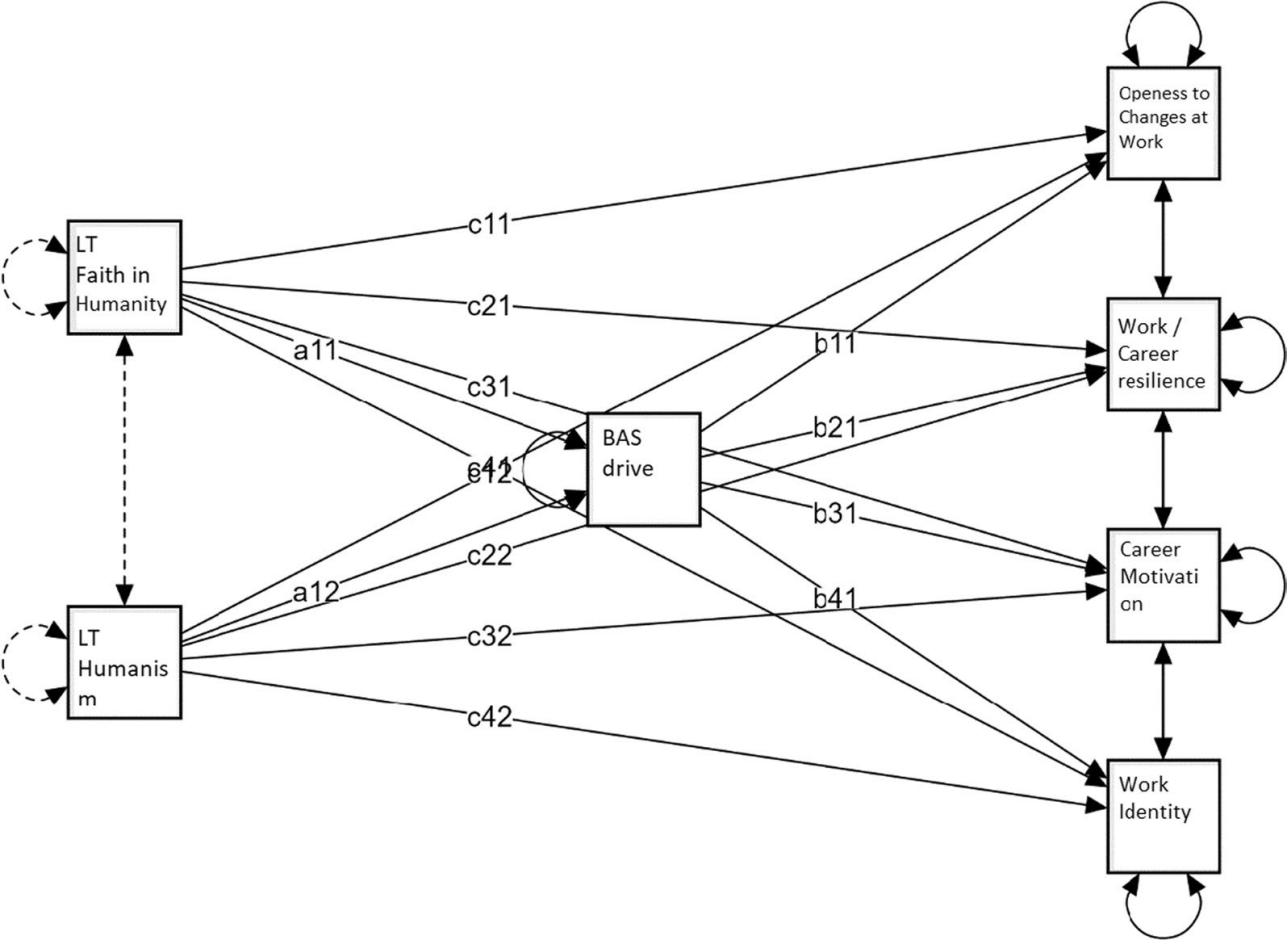
Light traits predict employability dimensions, with BAS drive mediation (a = direct effect; b = indirect effect; c = total effect)

**Fig. 3:**
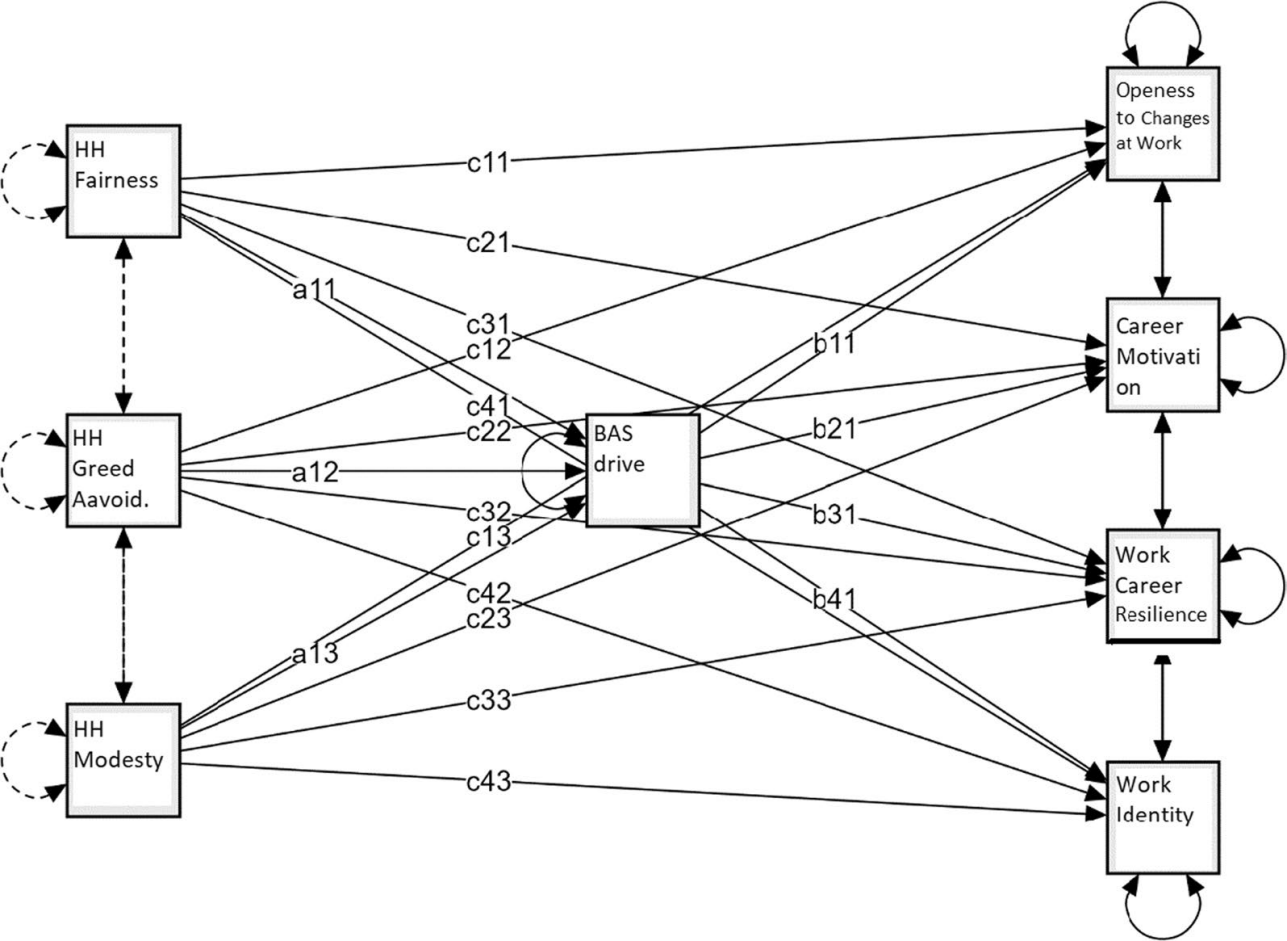
Honesty-Humility facets predict employability dimensions, with BAS drive mediation (a = direct effect; b = indirect effect; c = total effect)

Table 3 shows standardized estimates of the total, direct and indirect effects of BAS drive mediation on prosocial and antisocial personality traits in predicting employability dimensions. Figures 1, 2, 3 show the mediation models applied. In the mediation models dark traits (Fig. 1), light traits (Fig. 2), and honesty-humility facets (Fig. 3) are predictors of employability dimensions, with BAS drive as the mediator.

**Table 3:** Standardized estimates of total, direct and indirect effects of BAS drive mediation on prosocial and antisocial personality traits in predicting employability dimensions

	Direct effect	Indirect effect	Total effect	Mediation
*Openness to changes at work*				
DT Machiavellianism	− 0.077	0.011	− 0.066	
DT Narcissism	0.266***	0.110***	0.376***	Partial
DT Psychopathy	− 0.052	0.005	− 0.047	
HH Fairness	− 0.033	0.049**	0.017	
HH Greed avoidance	0.036	0.026	0.062	
HH Modesty	0.029	− 0.129***	− 0.100	
LT Faith in Humanity	0.174**	0.040*	0.214***	Partial
LT Humanism	0.079	0.036	0.115	
*Career motivation*				
DT Machiavellianism	0.076	0.020	0.096	
DT Narcissism	0.148*	0.201***	0.349***	Partial
DT Psychopathy	− 0.115	0.009	− 0.105	
HH Fairness	0.115*	0.067**	0.182***	Partial
HH Greed avoidance	− 0.007	0.035	0.028	
HH Modesty	− 0.221**	− 0.175***	− 0.396***	Partial
LT Faith in Humanity	− 0.061	0.073*	0.012	
LT Humanism	0.113	0.066	0.179*	
*Work/career resilience*				
DT Machiavellianism	− 0.123	0.021	− 0.102	
DT Narcissism	0.417***	0.209***	0.626***	Partial
DT Psychopathy	− 0.263***	0.010	− 0.253**	
HH Fairness	0.203***	0.078**	0.282***	Partial
HH Greed avoidance	− 0.131**	0.041	− 0.090	
HH Modesty	− 0.069	− 0.205***	− 0.273***	Full
LT Faith in Humanity	0.186***	0.074*	0.260***	Partial
LT Humanism	0.117	0.068	0.185*	
*Work identity*				
DT Machiavellianism	0.264***	0.017	0.282***	
DT Narcissism	0.252***	0.169***	0.421***	Partial
DT Psychopathy	− 0.344***	0.008	− 0.336***	
HH Fairness	0.141**	0.058**	0.199***	Partial
HH Greed avoidance	− 0.150**	0.030	− 0.120*	
HH Modesty	− 0.207**	− 0.152***	− 0.358***	Partial
LT Faith in Humanity	− 0.010	0.062*	0.053	
LT Humanism	0.167*	0.057	0.224**	

*p < 0.05; **p < 0.01; ***p < 0.001

BAS drive partially mediates the effect of narcissism in positively predicting openness to changes at work (standardized estimate of total effect = 0.376; SE = 0.078; z = 4.796; *p* < 0.001). Both narcissism and BAS drive explain 14% of the variance in openness to changes at work.

BAS drive partially mediates the effect of narcissism in positively predicting career motivation (standardized estimate of total effect = 0.349; SE = 0.078; z = 4.447; *p* < 0.001). Both narcissism and BAS drive explain 12% of career motivation variance.

BAS drive partially mediates the effect of narcissism in positively predicting work/career resilience (standardized estimate of total effect = 0.626; SE = 0.074; z = 8.498; *p* < 0.001). Both narcissism and BAS drive explain 39% of work/career resilience variance.

BAS drive partially mediates the effect of narcissism in positively predicting work identity (standardized estimate of total effect = 0.421; SE = 0.076; z = 5.530; *p* < 0.001). Both narcissism and BAS drive explain 17.7% of work identity variance.

BAS drive partially mediates the effect of HH fairness in positively predicting career motivation (standardized estimate of total effect = 0.182; SE = 0.053; z = 3.446; *p* < 0.001). Both fairness and BAS drive explain 3% of career motivation variance.

BAS drive partially mediates the effect of HH modesty in negatively predicting career motivation (standardized estimate of total effect = − 0.396; SE = 0.074; z = − 5.331; *p* < 0.001). Both modesty and BAS drive explain 15.6% of career motivation variance.

BAS drive partially mediates the effect of HH fairness in positively predicting work/career resilience (standardized estimate of total effect = 0.282; SE = 0.053; z = 5.364; *p* < 0.001). Both fairness and BAS drive explain 8% of work/career resilience variance.

BAS drive fully mediates the effect of HH modesty in negatively predicting work/career resilience (standardized estimate of total effect = − 0.273; SE = 0.074; z = − 3.702; *p* < 0.001). HH modesty through BAS drive explains 7.5% of work/career resilience variance.

BAS drive partially mediates the effect of HH fairness in positively predicting work identity (standardized estimate of total effect = 0.199; SE = 0.052; z = 3.821; *p* < 0.001). Both, fairness and BAS drive explain 4% of work identity variance.

BAS drive partially mediates the effect of HH modesty in negatively predicting work identity (standardized estimate of total effect = − 0.358; SE = 0.073; z = − 4.903; *p* < 0.001). Both modesty and BAS drive explain 12.8% of work identity variance.

BAS drive partially positively mediates the effect of faith in humanity in positively predicting openness to changes at work (standardized estimate of total effect = 0.214; SE = 0.066; z = 3.254; *p* = 0.001). Both LT faith in humanity and BAS drive explain 4.5% of the variance in openness to changes at work.

BAS drive partially mediates the effect of TL faith in humanity in positively predicting work/career resilience (standardized estimate of total effect = 0.260; SE = 0.064; z = 4.056; *p* < 0.001). Both faith in humanity and BAS drive explain 6.7% of work/career resilience variance.

## Discussion

Given that prosocial personality traits and self-regulation are among the employability skills sought by employers, the current study aimed to analyze anti/prosocial personality and self-regulation sources of dispositional employability. Narcissism is the most significant predictor of dispositional employability, together with low HH modesty. HH modesty correlates with DT more than any other HH facet [11], which may explain this. Narcissism tends to overuse character strengths, except modesty [71]. Narcissism positively predicted all employability dimensions and HH modesty predicted three. The results shed more light on the cultural universality of narcissism as a positive predictor of employability [36]. Partial and full mediation of BAS drive (motivation to follow one's goals) supported narcissism and low modesty predictions. On average it added 46% to the explained variance of predicted employability. The results confirm that a strong need for success, related to career exploration, is significant for narcissistic personalities [72].

But BAS drive was not a significant mediator for all the analyzed predictors. Psychopathy negatively predicted two employability dimensions (work/career resilience, work identity), and Machiavellianism positively predicted one employability dimension (work identity) [36]. BAS drive did not support these predictions. Neither did it support predictions by LT humanism. Psychopathy (callousness and impulsivity), the negative predictor of employability, is the opposite of LT humanism (valuing the dignity and worth of each individual). Showing respect for others predicts employability, regardless of goal achievement orientation. Workplace dignity can enhance employee satisfaction, engagement, and retention [73]. Respect for one’s dignity results from the notion that we are autonomous beings, capable of being ruled by self-given moral laws [74], in other words, be autonomous [75]. For people who are not driven by a goal but with higher levels of LT humanism, the workplace with respect for autonomy and dignity can be very attractive.

LT faith in humanity, with an average BAS drive contribution of 23.5%, predicted openness to changes at work and work/career resilience. In other words, believing in the fundamental goodness of humans, and goal orientation, predicts receptiveness, willingness to change, optimism about change, and future career. LT faith in humanity can be an effective trait for workers in demanding dynamic work environments (e.g., sales positions), where optimistic expectations relate to increased motivation and superior achievements [76], or in the academic field [77, 78]. The connection between optimism and achievement orientation is crucial here. People with high LT faith in humanity and goal orientation can be attracted to the work fields of high demands that require resilience and openness to change.

HH fairness (avoiding fraud and corruption), with an average BAS drive contribution of 31%, predicted career motivation (setting work/career-related goals), work/career resilience (optimism about future career), and work identity (defining oneself in terms of the organization, job, profession, or industry). As employers seek ethical behavior skills and moral integrity [2, 3, 79], it is crucial they focus on their attraction and retention tools. Employability plays a role in the voluntary turnover process. People high in employability have a lower turnover rate because they see more opportunities within the organization than do employees with low employability [1]. Regarding the current results, an ethically and morally integrated, goal-oriented employee is primarily focused on planning their future career. Therefore, such employees need support for their professional and personal growth and in planning their future careers. Nadelson [80] analyzed educational institutions that developed ethical and moral behavior in students. Besides other recommendations (like role modeling), she recommended developing ethical and moral behavior in students by fostering their personal growth. Akaah and Lund [81] pointed out that there is a significant relationship between organizational values and the ethical conduct of marketing employees.

Kantianism and HH sincerity did not predict employability. People who score high in HH sincerity are unwilling to manipulate others. Kantianism is defined as “treating people as ends unto themselves, not as mere means to an end”, which is the opposite of Machiavellianism [11] (p. 7). The tendency not to manipulate other people does not seem relevant in predicting employability, even in the context of behavioral activation towards goals. Similarly to Machiavellianism, HH sincerity and LT Kantianism appear irrelevant in employability predictions [36]. Thus, the current study contributes to existing knowledge on weak relationships between Machiavellianism and perceived employability that may be culturally universal.

### Practical implications

If we disregard narcissism, the dark traits (psychopathy, Machiavellianism) repeatedly predicted only one employability dimension – work identity. It relates to a genuine interest in others’ impressions [1]. If we disregard HH modesty, then prosocial traits (HH fairness, LT humanism) most often repeatedly predicted three employability dimensions: career motivation, work/career resilience, and work identity. Prosocial characteristics are more complex when it comes to predicting employability dimensions (with or without BAS drive contribution), and they affect more aspects of employability than simply dark traits. Nonetheless, their impact is not as strong as that of narcissism/low HH modesty.

Narcissism is typically found in grandiose self-promoters who continually crave attention [82]. People with a low HH modesty think they are superior and entitled to privileges that others do not have [83]. People with high narcissism and low modesty make a great impression on others [84, 85]. Their tendency to make a good impression may explain higher employability. Narcissism or low HH modesty, supported by BAS drive are the strongest predictors of employability. But it is not the narcissism or HH modesty that makes the self-presentation impressive. It is the skill of self-presentation and presentation skills that generally impress others. It is self-presentation that matters. Basic personal presentation, assertiveness, confidence, verbal presentation, and basic interpersonal and communication skills are included in individual factors – employability skills and attributes, according to McQuaid and Lindsay [15]. Guilbert et al. [14] recommend developing social, linguistic, and paraverbal skills that enable vulnerable people to behave adequately with their future employers. Self-presentation skills are known as one of the crucial antecedents of employability [24]. We suggest that for people with high modesty and low narcissism, developing self-presentation skills can increase their employability. Orientation toward self-presentation and goal achievement positively predicts employability dimensions.

To attract and retain ethical and moral employees, employers could use tools to encourage future career planning and personal and organizational growth [80, 81]. Developing learned optimism in prosocial young adults could aid adaptability to organizational changes [76]. These recommendations are valid in the context of personal goal orientation. Looking beyond BAS drive (activation toward goal), young people’s employability could be developed through respect and preservation of human dignity [73].

In the current research model, we examined all rather stable personality and employability factors, raising the question of how we could affect employability effectively. Although we examined the individual level of employability, employability is generally understood as the broader concept [15]. Broader concept of employability includes individual factors (employability skills and attributes, demographic characteristics, health and well-being, job seeking, adaptability, and mobility), personal factors (household circumstances, work culture, access to resources), and external factors (demand factors, enabling support factors). All dynamic parts of the employability system are open for development. For example, Guilbert et al. [14] define employability as “the possibility to access a suitable job or to remain employed, resulting from the dynamic and evolving interactions between governmental and educational policies, organizational strategy, individual characteristics, and the social, economical, cultural and technological context (p. 85).“ For employability development, all these contexts create opportunities. Our talents are nothing without effective ways of behavior, and everybody needs to develop employability skills in the time of huge technological, information, and environmental changes that society currently lives in. A high level of disposition can not save anybody without proper work and training. On the other side, working skills/experience contribute to predicting work performance, besides stable features [86]. The theory of social learning [87] can bring the inspiration to the development of dispositional defined employability models. The current study can point out the possible employability environments attractive to high/low goal-oriented people with specific dark/light profiles.

The results of the current study indicate that some of the behavioral and personality characteristics of dark traits, namely narcissism, can relate to positive outcomes, for example, in employability. The research revealed some positive features of narcissism too. Compared to psychopathy and Machiavellianism, narcissism overuses character strengths (apart from modesty). Underuse and overuse of character strengths can result in negative outcomes in addition to the socially valued positive aspects [71]. Narcissism is associated with a positive mental attitude as an indicator of pro-health behaviors [88]. People with high narcissistic trait claim to invest less effort in reaching their goals, although they invest not less but the same amount as other people do. In poor performance, they argue with a lack of effort rather than a lack of ability. They would benefit more from considering all objective feedback to improve their self-regulation [89]. But what lessons can we take from them? That it can be beneficial for us to be motivated by future aspirations than by past performance. It is good to invest more effort when the results are publicly evaluated, although some successful people say they invested only a small amount of effort.

### Future research implications

Even if employers do not seek dark traits in employees, some of the strategies adopted by dark personalities, underlined by goal orientation, are very successful in predicting employability. Generally, DTs predict work/career identity, focusing on making an impression on others. Narcissism predicted the most career/work resilience, which relates to an optimistic view of the future. Regarding these results, future analyses could focus on whether interventions in the form of strengthening self-image [90], self-presentation skills [91], presentation skills [92], or learned optimism [93] contribute to employability.

BAS drive contributed to the prediction of dispositional employability by HH fairness, HH modesty, LT faith in humanity, and narcissism. Behavior activation through BAS drive helps to increase employability where there is a tendency to attract attention and impress others (modesty and narcissism), avoid fraud and corruption (fairness), and to believe in the fundamental goodness of humans (faith in humanity) [11, 63, 83]. But it contributed most to predictions of employability by narcissism and modesty.

Other typical employability competencies include self-awareness [4], which can generally be developed through mindfulness [94]. Future research could explore the role of mindfulness in the employability of prosocial personalities. The use of mindfulness to foster university student employability [95] is based on the premise that developing coping strategies makes it easier for students to cope with job search stress and thereby enhances their employability. Mindfulness is a protecting factor against automated and maladaptive behavior and increases self-determined autonomous regulation [94]. Mindfulness also significantly mitigates aspects of emotional lability, such as neuroticism [96], and is recommended when training individuals with sensitive BIS [97]. The relationship between prosocial behavior and emotional lability is limited and inconsistent and has yet to be unequivocally confirmed [50]. For example, empathetic women focus more on BAS rewards, which is associated with prosocial behavior [98]. They have a lower BAS drive and seek to secure rewards through prosocial behavior, specifically empathy. Therefore, mindfulness could significantly contribute to employability, especially in prosocial personalities with high emotional lability, BIS, or low BAS drive.

### Limitations

The study has several limitations. Convenient sampling was used for the data selection process and so the results are not representative. The results are valid for the sample of Slovak university students investigated. Nonetheless, they align with those of researchers [36] who measured employability using different instruments on samples of different nationalities and support their validity. The research sample consisted of young adults at university. Although they may have had work experience, their focus was on their studies. Therefore, the results may be valid for students or graduates, but not undergraduates working/experienced adults, or adults without a university education.

We considered only two control variables (age and gender). However, many other variables that impact openness to organizational change may influence the examined associations, e.g., the need for achievement [99]. The need for achievement could have impacted the results, especially because the participation in the research was voluntary, which suggests that selection bias may be present. Peeters et al. [100] did not confirm any significant control variables among age, gender, educational level, contract type, or job level on perceived validity. Because of that, they reported results without control variables. In the next research, it would be beneficial to control the effect of various variables on dispositional employability.

The analysis did not include the employability facet of work/career proactivity because it had a non-standard distribution. The role of pro/antisocial traits and self-regulation in predicting work/career proactivity remains unclear. Some variables had lower internal consistency (e.g., modesty α = 0.55; ω = 0.57), although still higher than in the Slovak standardization sample (N = 1624; α = 0.53) [66]. Other research examining employability and dark traits [36] in Belgian, Swiss, and Togo samples faced these same issues.

The research design was cross-sectional. All the data were collected from the same sample at the same time. Given the novelty of research aim, we decided to focus on cross-sectional approach for obtaining initial results in an area with little or no empirical knowledge [101].

### Conclusion

Prosociality and self-regulation were shown to be significant predictors of dispositional employability. Despite not being required by employers, dark traits, namely narcissism, play a significant role in predicting employability. As a factor of self-regulation, BAS drive was the common source of dispositional employability, regardless of pro- or antisociality. Prosocial behavior significant for employability is showing respect to people, believing in the goodness of people, and avoiding fraud and corruption. The results provide higher education institutions and employers with an argument for developing self-presentation competencies in goal-oriented prosocial personalities. Employers could attract and retain goal-oriented prosocial employees by setting the right organizational values, providing learned optimism training, and supporting their personal and professional growth.

## Data Availability

The dataset generated and analyzed during the current study is available in the Figshare repository, https://doi.org/10.6084/m9.figshare.19286159. Research materials are available upon the references used in the methods section.
